# Presence of SARS-CoV-2 in urine is rare and not associated with acute kidney injury in critically ill COVID-19 patients

**DOI:** 10.1186/s13054-020-03302-w

**Published:** 2020-09-29

**Authors:** Robert Frithiof, Anders Bergqvist, Josef D. Järhult, Miklos Lipcsey, Michael Hultström

**Affiliations:** 1 Anesthesia and Intensive Care Medicine, Department of Surgical Sciences, Uppsala University, Uppsala, Sweden; 2 Clinical Microbiology and Infection Medicine, Department of Medical Sciences, Uppsala University, Uppsala, Sweden; 3 Clinical Microbiology and Hospital Infection Control, Uppsala University Hospital, Uppsala, Sweden; 4 Zoonosis Science Center, Department of Medical Sciences, Uppsala University, Uppsala, Sweden; 5 Hedenstierna Laboratory, CIRRUS, Anesthesiology and Intensive Care, Department of Surgical Sciences, Uppsala University, Uppsala, Sweden; 6 Integrative Physiology, Department of Medical Cell Biology, Uppsala University, Uppsala, Sweden

**Keywords:** Acute kidney injury, Critical illness, COVID-19, SARS-CoV-2, Urine

Dear Editor,

Patients infected with SARS-CoV-2 requiring intensive care due to coronavirus disease 2019 (COVID-19) frequently develop acute kidney injury (AKI) [1], but the underlying mechanisms are poorly explored. SARS-CoV-2 has been found in both urine and the kidneys, where it has been suggested to cause proximal tubule damage [2–4]. Direct renal infection of SARS-CoV-2 causing AKI potentially leads to viral shedding in urine. However, to our knowledge, no study has been undertaken to investigate urinary levels of SARS-CoV-2 in patients with AKI.

In this report, SARS-CoV-2 RNA levels were prospectively investigated in urine of patients with upper or lower airway swab test PCR-verified COVID-19, admitted to a Swedish intensive care unit (ICU, *n* = 81). The presented data is part of a study approved by the National Ethical Review Agency (2020-01623). Informed consent was obtained from the patient or next of kin. The Declaration of Helsinki and its subsequent revisions were followed. Nucleic acid was extracted from urine samples using NucliSENS® eMAG® (bioMerieux), and the amount of viral RNA was quantitated by detection of SARS-CoV-2 E and N-genes using real-time RT-PCR according to previously described protocols [5, 6]. For quantitative assessment, the assay was calibrated against a synthetic RNA standard from ATCC and the detection limit was determined to 200 copies/ml.

SARS-CoV-2 was found in urine of only 6 patients (7%). The median concentration was 1200 copies/ml (range 300–2800). Urinary viral secretion was not associated with mortality or severity of disease as estimated by Simplified Acute Physiology Score 3 (SAPS3) on admission, length of stay in the ICU, the need for invasive ventilation, or renal replacement therapy (Table 1). Based on changes in plasma creatinine, 51 (63%) patients developed AKI during their ICU stay. Only 5 (10%) of those patients had detectable SARS-CoV-2 RNA levels in the urine. This indicates that urinary secretion of SARS-CoV-2 is uncommon in COVID-19-associated AKI. Furthermore, detection of SARS-CoV-2 RNA in urine was not significantly associated with renal dysfunction and was most frequent in the mildest stage of AKI (Table 1). Of interest is that positive samples in patients with AKI were collected significantly further from onset and peak AKI as compared to negative samples (Table 1).

**Table 1:** Patient characteristics and ICU treatment of 81 patients admitted to intensive care due to severe COVID-19 divided by findings of SARS-COV-2 in urine or not. Values are represented as median (IQR) or *n* (%). Data for “SAPS3” and “Days between onset of symptoms and sampling” were missing for one and 4 patients, respectively, in the group negative for SARS-COV-2 in urine. The *p* value originates from the Mann-Whitney *U* test for continuous parameters and the chi-square test for categorical parameters. Values are represented as median (IQR) or *n* (%). *p* < 0.05 is considered significant. *AKI* acute kidney injury

	SARS-COV-2 urine negative	SARS-COV-2 urine positive	*p*
	(*n* = 75)	(*n* = 6)	
Gender, fe*male*, *n* (%)	17 (23)	2 (33)	0.57
Age, *years*	61 (53–70)	58 (39–69)	0.59
Days between onset of symptoms and sample	15 (12–16), *n* = 71	14 (12–16)	0.68
Days between ICU admission and sample	4 (3–4)	4 (4–5)	0.33
SAPS3 on admission	53 (47–57), *n* = 74	57 (44–60)	0.70
ICU-free days	14 (0–19)	22 (5–22)	0.26
Ventilator-free days	20 (0–28)	27 (6–28)	0.50
Renal replacement therapy-free days	28 (0–28)	28 (7–28)	0.95
ICU mortality, *n* (%)	13 (17)	2 (33)	0.37
30-day mortality, *n* (%)	14 (19)	2 (33)	0.41
Acute kidney injury, *n* (%)	46 (61)	5 (83)	0.28
Stage I	23 (31)	4 (67)	0.07
Stage II	9 (12)	0 (0)	0.36
Stage III	14 (19)	1 (17)	0.90
Days between onset of AKI and sample	2 (− 1–4), *n* = 46	6 (5–8), *n* = 5	0.01
Days between peak *P*-creatinine and sample	− 2 (− 7–3), *n* = 46	5 (4–5), *n* = 5	0.03

Limitations of the present study include that urine was not sampled repeatedly in the same patient. In case of varying viral secretion, this may have led to an underestimation of the number of patients being positive prior to or during the complete ICU stay. Furthermore, AKI was determined based only on the change in plasma creatinine, not taking into account urine output. As AKI stages are defined by the maximum change of either plasma creatinine or urine output, we may have underestimated the incidence of AKI in this cohort. Finally, the low concentration of viral RNA in a limited number of patients prevents definitive conclusions regarding mechanisms of viral urinary secretion. A late onset of viral shedding in the urine may suggest a slowly developing glomerular filtration barrier dysfunction, but future studies are needed to investigate this in detail.

Here we show that urinary secretion of SARS-COV-2 is scarce in critically ill COVID-19 patients. In this cohort, SARS-CoV-2 RNA was not more frequently detected in urine of patients that died or developed acute kidney injury. This suggests that determining viral presence in urine will not aid in predicting or grading renal dysfunction or severity of disease in COVID-19. Our findings do not support direct renal SARS-COV-2 infection as an important mechanism of COVID-19-induced AKI, since renal infection likely would result in viral shedding in urine and thus a higher frequency of PCR positivity in urine of patients with AKI.

## Data Availability

Data in the current study is available from the corresponding author on a reasonable request.
